# Mentalization-based intervention for parents with elevated parenting stress: protocol of a RCT of the lighthouse parenting programme in China

**DOI:** 10.3389/fpsyg.2026.1728608

**Published:** 2026-05-08

**Authors:** Ruoshi Hu, Svenja Taubner, Anna Georg

**Affiliations:** 1 Psychological Institute, Department of Clinical Psychology and Psychotherapy, University Heidelberg, Heidelberg, Germany; 2 Institute for Psychosocial Prevention, Centre for Psychosocial Medicine, Heidelberg University Hospital, Heidelberg, Germany

**Keywords:** cultural adaptation, lighthouse parenting programme, mentalization-based parenting intervention, parenting stress, prevention of early mental health

## Abstract

The period of childhood and early adolescence, specifically prior to the age of 14, is a critical phase for the prevention of mental health problems. Nevertheless, prevention programs for this age group remain under recognized in China. In this domain, the lighthouse parenting programme (LPP) has successfully achieved initial results in several countries. This paper describes the protocol of a randomized controlled trial (RCT) using a waiting list control design to evaluate the potential efficacy and applicability of LPP with Chinese parents. The trial is designed as an exploratory investigation into possible associations between the LPP and changes in parenting stress, parenting-related psychological correlates, and the mental health risk profiles of their children.

**Methods:**

A total of *N* = 44 parents with elevated parenting stress and raising children aged 4 to 14 years will be recruited. Participants will be randomly assigned in a 1:1 ratio to either the treatment immediate group (TI) or a waitlist control group (WL). Primary outcomes will include participant's parenting stress and other parenting-related correlates (e.g., parental mentalizing, personality functioning, self-efficacy, and mental health, parent-child relationship quality), and children's variables (e.g., child's behavior difficulties and temperament). Parents' subjective experiences and satisfaction with the intervention will also be assessed. Secondary outcomes will include recruitment, retention, and dropout rates, as well as participant characteristics and factors influencing intervention effectiveness. The data will be analyzed qualitatively and quantitatively.

**Discussion:**

This study signifies the first implementation of LPP in Asia, emphasizing prevention. It seeks not only to evaluate the clinical utility of LPP but also to initially explore the implementation potential of MBT-based prevention programs in China. Findings from this trial are expected to inform future directions in the development and implementation of culturally attuned psychotherapeutic interventions in China.

**Clinical trial registration:**

clinicaltrials.govclinicaltrials.gov, identifier: NCT06858059.

## Introduction

Emerging evidence suggests that the period before age 14 represents a critical window for the prevention of mental health difficulties (Kessler et al., 2005). Despite this, systematic approaches to identifying at-risk children and delivering timely, targeted interventions remain limited in routine clinical practice (Plueck et al., 2010; Heinrichs et al., 2005; Bolinski et al., 2022). Behavioral problems in children with such risks often emerge early and exert a significant burden on parenting. These difficulties frequently elicit high levels of parenting stress. Developmentally, children in this age group are typically not equipped to seek support autonomously, underscoring the importance of facilitating intervention access through parents. Enhancing parental access to support thus represents a critical strategy for the timely prevention of early mental health issues.

Moreover, it has been found that caregivers' own psychological vulnerabilities and parenting-related correlates are not only strongly associated with children's early mental health problems, related behavioral problems, and competence development through parenting stress (Ribas et al., 2024), but also frequently engage in a vicious cycle: children of parents with high levels of parenting stress are at greater risk of developing such problems, which in turn further elevate the parents' parenting stress (Evers et al., 2023; Cherry et al., 2019; Dijk et al., 2022; Daundasekara et al., 2021; Huang et al., 2025; Kochanova et al., 2022). Moreover, the association between children's externalizing behaviors—such as emotion dysregulation, aggression, and attention problems—and parenting stress is significantly stronger than that observed with internalizing problems, thereby positioning externalizing behavior problems as a more salient influencing factor within this dynamic (Cooke et al., 2019; Williford et al., 2007).

Existing research on parenting stress among Chinese parents remains limited. Preliminary evidence indicates that parents report high levels of stress stemming from time constraints, unmet guidance needs and work–life conflicts (Department of Chinese Women Children's Federation, 2023; Bian et al., 2022; Qian et al., 2021; Lau and Power, 2020). The influence of parenting stress on parenting behavior is also evident in Chinese families, manifesting as harsh discipline (Niu et al., 2018). Furthermore, paternal parenting stress has been found to positively predict parental burnout and children's emotional–behavioral problems (Yue et al., 2025), whereas higher parenting self-efficacy is associated with a lower likelihood of falling into high-stress groups (Wang and Cheng, 2025). Collectively, these findings suggest that Chinese parents lack effective and contextually appropriate parenting strategies, underscoring a pressing need for targeted interventions. However, relevant intervention programs remain scarce (Qu et al., 2024). In this context, introducing well-established programs from other regions represents a viable first step toward addressing this practical gap.

Parent-focused interventions have shown preliminary efficacy in reducing parenting stress, enhancing parental wellbeing, and improving parenting behaviors—including reduced harsh discipline or coercive parenting practices and increased positive discipline and encouragement. These improvements may, in turn, contribute to a reduction in parent–child conflict. Furthermore, children's externalizing problems (e.g., aggression, hyperactivity) and internalizing problems (e.g., anxiety, depression) are reduced (Bjørknes and Manger, 2013; Gervinskaite-Paulaitiene et al., 2023; Scott et al., 2014). Based on these findings, researchers have continued to investigate potential “meta-factors” that may influence this process. Among various mechanisms of change, the enhancement of parental reflective functioning and mentalizing capacity has garnered significant attention and is recognized as a core mechanism linking parent-level improvements to child behavioral outcomes (Barlow et al., 2021; Lavender et al., 2023; Nieto-Retuerto et al., 2024). This improvement is particularly marked within the domain of mentalization-based parenting interventions.

Mentalization-based therapy (MBT), widely used in reducing personality disorder symptomatology (Luyten et al., 2020), has demonstrated increasing relevance in prevention in the parenting context (Byrne et al., 2020; Sadler et al., 2013; Sleed et al., 2013). Initial evidence supports the assumption that an increased parental understanding of their own and their children's internal states, fosters more adaptive and sensitive caregiving responses in parental challenges and improves parent–child relationships (Barlow et al., 2021). MBT is also thought to reduce the intergenerational transmission of maladaptive attachment patterns (Midgley, 2024). Conversely, MBT remains relatively novel and underexplored within the Chinese context. To date, only one published study has examined its application, specifically evaluating mentalization-based family therapy (MBFT) combined with mindfulness therapy to reduce suicidal ideation in adolescents (aged 12–18) with depressive disorders in a hospital setting (Fan et al., 2024).

By contrast, MBT has already been implemented relatively widely in other regions. The lighthouse parenting programme (LPP; Byrne et al., 2015) is currently one of the most widely implemented MBT parenting intervention and has to date been adopted across multiple countries in Europe and South America (Byrne et al., 2019; Volkert et al., 2022; Hestbaek et al., 2026; Gervinskaite-Paulaitiene et al., 2023; Costa-Cordella et al., 2025). With a strong emphasis on attachment theory and child development, LPP explicitly targets parental reflective functioning, promotes epistemic trust, and aims to support parents in exploring their own emotional experiences while responding to their children's needs. Initial evidence supports the assumption with observed changes in a range of domains, including reductions in child maltreatment, coercive parenting, parenting stress, and child behavioral problems, along with improvements in parental mental health, family relationships, parental teamwork, self-efficacy, self-focused mentalizing, and parenting conditions of parents with psychological disorders (Byrne et al., 2019; Gervinskaite-Paulaitiene et al., 2024; Byrne, 2020; Hestbaek et al., 2025). However, these preliminary findings are predominantly derived from pilot investigations or pre–post designs, often characterized by limited sample sizes and constrained statistical power. To date, randomized controlled trials evaluating the effectiveness of the LPP remain limited. Existing evidence consists of an RCT of the 20-week LPP conducted in England, along with a cluster of related trials in Germany—most notably, an RCT of the 5-week LPP carried out within the UBICA project (Neukel et al., 2021; Sleed et al., 2023; Herpertz et al., 2025). Furthermore, its application and empirical evaluation within Asian populations remain entirely absent from the extant literature.

Despite its promise, LPP has yet to be evaluated in non-Western cultural contexts. As the first application of the LPP in Asia, this study addresses a significant gap in research on interventions for parents experiencing elevated parenting stress. In terms of efficacy, the study will explore the extent to which the LPP may influence on parents' self-assessed results of participant's parenting stress and other parenting-related correlates (e.g., parental mentalizing, personality functioning, self-efficacy, and mental health), parent-child relationship quality, and children's variables (e.g., child's behavior difficulties and temperament), alongside parents' subjective experiences and satisfaction with the program. These outcomes, particularly the qualitative findings, will inform the ongoing refinement of the LPP with respect to its cultural relevance and applicability within Eastern contexts.

Additionally, as the first prevention program for parents with elevated parenting stress in China, the study will evaluate the applicability of LPP implementation. This includes examining recruitment rates, participant characteristics, retention, dropout, and adherence, as well as reasons for attrition and possible variables that might be associated with intervention effectiveness. The findings aim to inform the development of scalable, context-sensitive early intervention strategies for preventing child mental health difficulties in China.

### Primary research questions

To evaluate the potential efficacy of the randomized controlled trial (RCT), the primary research questions focus on changes in parenting stress:

- Do parents in the TI group report a greater reduction in parenting stress index (PSI) scores following the 5-week intervention, compared to those in the WL group?

### Secondary research questions

Firstly, to more comprehensively investigate the mechanisms through which the LPP alleviates parenting stress, additional correlates related to parents, children, and the parent–child relationship, as well as participants' experiences with and satisfaction toward the program, are assessed before and after the intervention. Secondly, in the context of implementing the LPP in China, exploratory questions arise regarding its preliminary feasibility and practical applicability. On this basis, the present study is designed to address the following secondary research questions:

- Do parents in the TI group report greater improvements in parenting-related correlates—including parental reflectivity, self-efficacy, mental health, and the quality of the parent-child relationship—compared to the WL group following the 5-week intervention?
- Do parents in the TI group report greater improvements in child outcomes, specifically reductions in behavioral difficulties and positive changes in temperament (e.g., emotionality), compared to the WL group post-intervention?
- How do parents subjectively experience their participation in the LPP?
- What is the overall level of participant satisfaction with the intervention program?
- What are the recruitment rates?
- What are the characteristics of study enrollees and participants?
- What are the retention, dropout, and compliance rates?
- What are the reasons for participant dropout?
- What factors might be associated with the retention and effectiveness of the intervention?

## Method

### Design

This study is a single-center, randomized controlled trial using a waitlist control condition assessing the first implementation of LPP in China. The objective of this study was to preliminarily explore the effectiveness and feasibility of LPP in addressing parenting stress among Chinese parents.

**Figure 1:**
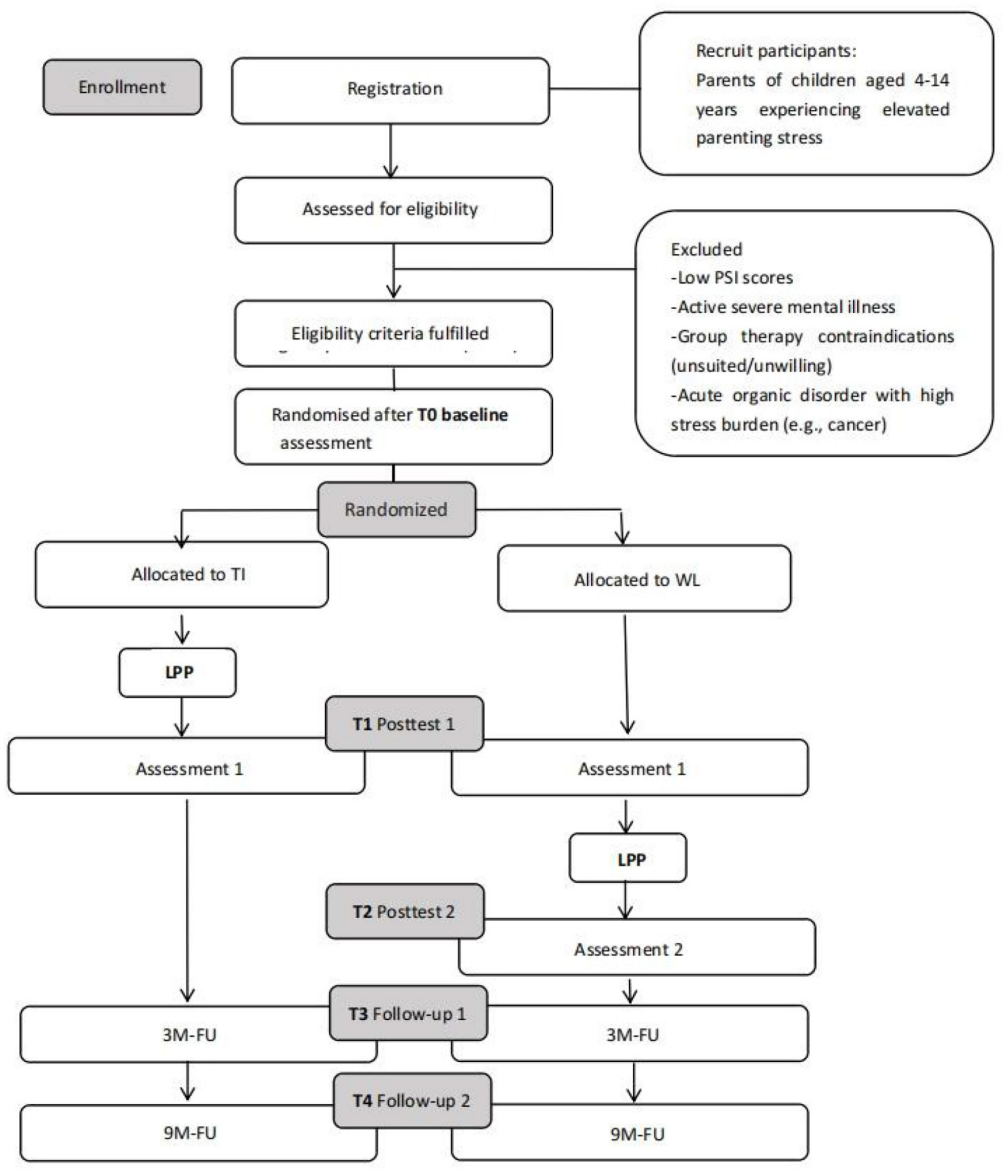
Expected flow of participants from recruitment through to the end of the study.

The study will be conducted at a comprehensive hospital in Guangzhou, China. Parents who are enrolled in the study will participate in a 5-week LPP intervention. Parents will be asked to complete self-report questionnaires and to participate in a follow-up at 3 months and 9 months after study inclusion. The study flow is illustrated in Figure 1.

### Recruitment

The subject recruitment strategy employed in this study is multifaceted, encompassing targeted referrals, publicizing lectures, printed advertisements (flyers and posters), and social media. Potential participants will be furnished with pertinent information, including a link to the program profile and an enrollment link. All parents will be required to contact the project team through self-enrollment.

### Inclusion and exclusion criteria

The study further aims to examine whether the LPP, in addition to reducing parenting stress, may exert a preliminary preventive effect on children's mental health risk. As the optimal age for prevention falls below 14 years, this cutoff was applied. To balance heterogeneity of developmental characteristics, measurement coverage and feasibility of parental participation, the child age range was set from 4 to 14 years. Eligible participants are parents of children aged 4 to 14 years experiencing elevated parenting stress, defined as a Parenting Stress Index (PSI) score ≥ 85 (critical high level) with informed consent. Exclusion criteria included: (1) serious physical illness (e.g., cancer), (2) active severe mental illness, and (3) communication difficulties or language barriers that would prevent meaningful participation in a group-based intervention.

### Sample size

The determination of these parameters was facilitated by the utilization of the PASS 15.0 (NCSS Statistical Software, East Kaysville, UT, USA) software. Preliminary studies in the field have indicated a decline in the values of the indicators—total stress of PSI in the LPP intervention group, with a mean decrease of 11.3, and a corresponding decrease in the control group, with a mean decrease of 3.1 based on a previous MBT-based early intervention study (Fonagy et al., 2016). The experimental design employed a parallel approach, with the groups assigned in a 1:1 ratio. Given the exploratory nature of this study and resource constraints, a relatively liberal alpha level of 0.1 was adopted for sample size estimation. The clinical threshold of non-inferiority was set at 10% and the power level was set at 0.8. PASS15.0 calculated a total of 20 cases for each group. In the preparatory phase for this RCT, a pilot trial was conducted with a small cohort of five parents. As a zero dropout rate was observed in this pilot, no oversampling to compensate for participant attrition was deemed necessary. However, based on attrition rates observed in a comparable Chinese trial and accounting for the waitlist-control design, we conservatively estimated an attrition rate of approximately 10% (Jiang et al., 2024). Consequently, a minimum total sample size of 44 participants (22 per group) was determined to be required.

### Randomization

Randomization will occur immediately following the completion of baseline assessment. Eligible participants will be assigned in a 1:1 ratio to either the immediate intervention group or a waitlist control group using a computer-generated sequence with random block sizes of 4 and 6. Stratification was not performed given the exploratory nature of the study and the anticipated sample size. An independent data manager generated the allocation sequence, and participant enrollment was conducted by a clinical interviewer, who then accessed a secure web-based system to obtain the treatment assignment. Baseline comparability between conditions will be examined on key demographic and clinical variables. Participant blinding is not feasible, as individuals must be informed whether they will begin the intervention immediately or after a 2-month delay.

### Intervention

The lighthouse parenting programme (LPP) implemented in this study is a revised version developed by the Institute for Psychosocial Prevention, University Hospital Heidelberg (Taubner et al., 2019), consisting of five weekly group sessions and five accompanying individual sessions. Group sessions form the core of the intervention, introducing key mentalization-based treatment (MBT) concepts—such as mentalization, emotional triggers, autopilot behavior, and intergenerational transmission of attachment—through metaphors drawn from a sea voyage. These sessions foster reflection, shared understanding, and collaborative problem-solving around parenting challenges. Individual sessions are integrated with group content and tailored to each parent's needs, offering focused exploration of personal patterns in parenting and the influence of their family of origin. Parents are supported in observing and mentalizing their own and their child's behaviors, while developing strategies for change and resource access. Group sessions are facilitated weekly by a consistent, experienced psychotherapist, who also delivers the individual sessions. The 5-week program delivers one group and one individual session per week.

## Measures

### Primary outcomes

#### Parenting stress

The parenting stress index/short form (PSI/SF; Abidin, 1986) assesses self-reported PS with 36 items. Items are rated on a five-point Likert scale from 1 (strongly disagree) to 5 (strongly agree). The Chinese version is consistent with the original structure and the internal consistency of Chinese version of the total scale was 0.93, with 0.85 for Parental Distress scale, 0.86 for Parent-Child Dysfunctional Interaction scale, and 0.87 for Difficult Child scale (Ren, 1995).

### Secondary outcomes

(1) Additional parent-, child-, and parent–child relationship-related correlates.

#### Child behavior difficulties

Strengths and difficulties questionnaire (SDQ; Goodman, 1997) assess five sub-scales (hyperactivity, emotional, conduct problems, problems with peers and prosocial), each of them includes five items. The questionnaire asks parents to rate their child's emotional and behavioral problems on a 3-point Likert scale (ranging from 0 = not ture to 2 = certainly true). The Cronbach's α of Chinese version were ranging between 0.30 and 0.83. The reliability was 0.40–0.79 (Du et al., 2008).

#### Temperament of child

Emotionality, activity and sociability temperament inventory (EAS; Buss and Plomin, 1986) includes three scales that assess parents' perceptions of their children's emotionality, activity level, and sociability. It is composed of 20 items, rated on 5-point scale anchored by 1 (not typical of child) and 5 (very typical of child). It was demonstrated the adequacy of parent perceptions as general valid measures of children's temperament (Slabach et al., 1991; Porter et al., 2005).

#### Parental mentalizing

Parental reflective functioning questionnaire (PRFQ; Luyten et al., 2017) assesses three scales with 18 items: (1) interest and curiosity in mental states (IC), (2) certainty of mental states (CMS), and (3) prementalizing (PM), which signifies deficits in parental reflective functioning (PRF). Items are rated on a 7-point Likert scale ranging from “strongly disagree” to “strongly agree.” The three-factor model of Chinese vision had a good fit and criterion-related validity (Ye et al., 2022).

#### Parental self-efficacy

Brief parental self-efficacy scale (BPSES; Woolgar et al., 2023) is a 5-item scale, which measures how confident parents are that they can manage the tasks and demands of parenting. Each item on a five-point Likert scale: strongly disagree; disagree; neutral; agree; strongly agree (corresponding to scores 1 to 5, respectively). This study marks the first administration of this questionnaire in China. A Chinese version was translated for this purpose and demonstrated good reliability.

#### Parental personality functioning

Level of personality functioning scale-brief form 2.0 (LPFS; Weekers et al., 2019) is a 12-item scale. Each item is rated on a 4-point scale from 1 (very false or often false), 2 (sometimes false or somewhat false), 3 (sometimes true or somewhat true) to 4 (very true or often true). It assesses personality functioning, with higher scores indicating more impaired personality functioning. The Chinese version of this scale had good reliability (Cheng et al., 2025).

#### Parental mental health

The patient health questionnaire is a DSM-IV-based screening tool for the diagnosis of mental disorders. This study used a 9-item depression module (PHQ-D) and a 7-item generalized anxiety module (GAD-7). Participants rated the frequency of symptoms over the past 2 weeks on a 4-point Likert scale (1 = not at all, 4 = much more than usual). The Chinese version of the scale was reported to have good reliability (Yu et al., 2012).

#### Parents–child relationship quality

The multiperspective parent–child relationship questionnaire (M-PCR; Müller and Achtergarde, 2018) contains 14 items for each rater on a five-point Likert scale (0 = I disagree; 4 = I agree totally) and provides subscale scores for the contents of affective bond and functional-conflict, which are aggregated in the main M-PCR score. As no Chinese version of this questionnaire existed, it was translated and revised for the present study. The translated version demonstrated excellent reliability, and its use was formally approved by the original developers.

#### Satisfaction

Questionnaire to measure patient satisfaction-8 (ZUF-8 Questionnaire; Schmidt and Nübling, 2002) is a method of documenting overall patient satisfaction at the conclusion of hospitalization. Each of the eight items is scored on a 4-point scale (1 = poor, 4 = excellent), four of which are reverse scored and summed to form a total scale with a total score of 32. The instrument is frequently utilized in surveys assessing satisfaction with interventions. It is also frequently used in surveys related to satisfaction with the quality, contents, and effects of the intervention. The internal consistency (Cronbach's alpha) was between 0.87 and 0.93 for the individual samples and 0.92 for the total sample of the above-mentioned routinized patient surveys (*n* = 6,865). The values for split-half reliability and parallel test reliability were equally high.

Table 1 shows a detailed outline of the planned measures at each follow-up point throughout the trial.

**Table 1:** Schedule of assessment administration for a randomized controlled trial of LPP.

Construct	Instrument	Items	Pre-test	Post-test	Follow-up
Parental stress	Parenting Stress Index-Short Form (PSI-SF)	36	×	×	×
Child behavior difficulties	Strengths and difficulties questionnaire (SDQ)	25	×	×	×
Temperament of child	Emotionality, activity and sociability temperament inventory (EAS)	20	×	×	×
Parental mentalising	Parental reflective functioning questionnaire (PRFQ)	18	×	×	×
Parental self-efficacy	Brief parental self-efficacy scale (BPSES)	5	×	×	×
Parental personality	Level of personality functioning scale-brief form 2.0 (LPFS)	12	×	×	×
Parental mental health	Brief patient health questionnaire (Brief PHQ)	16	×	×	×
Parents–child relationship quality	Multiperspective parent–child relationship questionnaire (M-PCR)	18	×	×	×
Satisfaction	Questionnaire to measure patient satisfaction-8 (ZUF-8)	8		×	

#### Subjective experience of participation

One-to-one semi-structured interviews will be conducted to explore the subjective experience of the LPP. The interviews was employed in a similar intervention project in Germany (Georg et al., 2022), the subsequent questions explored the following topics utilizing open-ended and closed-ended questions: expectations before treatment, the therapeutic relationship, interventions and significant moments, outcomes and termination of therapy. After each question, parents will be encouraged to give examples or mention specific situations to illustrate their experiences. Further probes will be used by the interviewers to clarify ambiguities.

(2) Recruitment rates for each recruitment methods, retention rates, dropout rates, compliance rates, etc.

**Recruitment, retention, and intervention completion**: the proportion of parents screened relative to those enrolled; the proportion of enrolled parents who completed the intervention; dropout rates; and reported reasons for attrition.

**Program compliance**: session attendance, including the number of group and individual sessions attended per participant.

**Influencing factors**: factors of retention and effectiveness of the intervention.

### Assessment of treatment fidelity

The project leader, a researcher in the IPP, specializing completed formal LPP training and participated in multiple intervention sessions in Germany. Prior to the implementation of the program in China, the project team conducted a cultural adaptation of the LPP (Hu et al., in prep), with ongoing supervision provided by the original German LPP team. Based on the results of thematic analysis of data collected from community stakeholders, cultural adaptation was further explored using an integrative theoretical framework combining the cultural adaptation process model (comprising preparation, collaborative implementation, and iterative refinement) and the ecological validity model (including language, persons, metaphors, content, concepts, goals, methods, and context). While certain peripheral components of LPP were adapted to align with the needs and realities of the new target group, the core components—enhancing parents' mentalized understanding of their children's emotions and breaking the intergenerational transmission of attachment trauma—were consistently retained and emphasized. Following cultural adaptation, the program was tailored to the daily circumstances of Chinese parents to minimize any sense of distance from an intervention originating in a different cultural context. For instance, childhood objects used to prompt recall of early experiences were adapted: items unfamiliar to Chinese parents were replaced with culturally meaningful counterparts from their own childhoods, while retaining the original symbolic significance. To ensure treatment fidelity, all intervention sessions will be video-recorded and reviewed as part of regular supervision.

### Data collection procedure

Subsequent to enrollment and the provision of informed consent, parents will be invited to the site where the intervention will be administered to participate in the questionnaire assessment and interview on-site (T0). Parents eligible to participate will then be randomly assigned to either the treatment immediate group (TI) or the waitlist control group (WL). Participants in the WL group were offered to participate in the intervention after a 2-months observation period. During this 2-month period, TI group received the full intervention and completed the posttest at the conclusion of this period. Prior to the WL intervention, participants in both groups will receive a second questionnaire assessment (T1). Subsequent to the WL's completion of the intervention, a third questionnaire assessment will be conducted (T2). At subsequent follow-ups, both groups will continue to complete two assessments (T3 and T4) at 3- and 9-months post-intervention.

### Statistical analysis

All parents assessed at baseline (T0) will be included in the data analysis following intent-to-treat principles. Sociodemographic variables and baseline assessment data will be used to characterize the enrolled sample. Descriptive data on recruitment, dropout, and retention rates will also be collected. Additionally, qualitative data—including reasons for dropout—will be analyzed to evaluate the appropriateness and acceptability of the study procedures and intervention. The primary objective of this study is to examine changes in participant's parenting stress and other parenting-related correlates (e.g., parental mentalizing, personality functioning, self-efficacy, and mental health), parent-child relationship quality, and children's variables (e.g., child's behavior difficulties and temperament) before and after the intervention, measured by standardized questionnaires. Participant satisfaction will be assessed using a standardized questionnaire. Furthermore, parents' subjective experiences during the LPP will be explored through semi-structured interviews. The quantitative data will be analyzed using SPSS 21.0 (IBM Corp., Armonk, NY, USA), employing repeated measures ANOVA (rmANOVA), *t*-tests, and chi-squared tests. The analysis of qualitative data will be conducted using the grounded theory methodology.

### Patient and public involvement

Neither patients nor the public are directly involved in the design, choice of outcome measures or recruitment for the study.

## Discussion

In light of the pronounced discrepancy between the pressing demand for and the prevailing scarcity of effective, culturally suitable psychological interventions for parenting among Chinese parents, the LPP, as an approach for which emerging evidence suggests potential efficacy, was implemented for the first time in China. This protocol outlines a pilot study evaluating the effectiveness and applicability of the LPP in parents experiencing elevated parenting stress. The primary aim of the program is to enhance parental mentalizing capacities through a focus on parent–child interactions. Implementation of LPP has been associated with reductions in parenting stress and difficulties, improvements in parent–child relationship quality, and decreased risk of mental health problems in children from higher-risk families. While the effectiveness of MBT in enhancing mentalizing, and of LPP in addressing parenting challenges, has shown promise in several Western countries (Volkert et al., 2022; Gervinskaite-Paulaitiene et al., 2023, 2024; Byrne et al., 2019, 2020; Hestbaek et al., 2025), empirical evidence from China remains scarce. Although we anticipate positive outcomes, it remains unclear how the effects of the program may vary across cultural contexts, underscoring the need for systematic investigation in the present study.

Despite the growing international consensus on the critical importance of prevention in mental health (Kessler et al., 2005; Plueck et al., 2010; Bolinski et al., 2022), this concept is still emerging and underexplored in the Chinese context. This study seeks to address that gap by recruiting participants for an prevention-focused LPP trial and examining participant characteristics, program adherence, and dropout. The findings will inform the cultural adaptation of prevention strategies and MBT-based interventions in China, contributing to the broader development of early mental health prevention frameworks in non-Western settings.

Nonetheless, several limitations of the current approach merit attention. Given that this is an initial pilot study conducted under constrained conditions, the randomized controlled trial will be implemented with the minimum necessary sample size, thereby resulting in a relatively small cohort whose findings can only offer preliminary implications. Thus, the results should be interpreted with caution and await replication in future studies employing larger samples. Furthermore, because many child outcome measures are age-specific, the age range of eligible children was restricted to 4–14 years to ensure consistency in assessment tools, thereby excluding parents of younger children. Additionally, while all measures are considered appropriate for use across the full 4–14 age range, supporting meaningful statistical analyses, the substantial heterogeneity inherent to different developmental characteristics may be associated with variations in parenting challenges and the course of improvement. More nuanced differences will be examined through the qualitative strand of this study. Future adequately powered research could target narrower age bands, facilitating a more integrated interpretation of qualitative and quantitative findings and enabling conclusions that better reflect developmental specificity at each key stage between the ages of 4 and 14.

Owing to practical constraints of the study, all outcome measures rely on self-report instruments, which may be subject to reporting bias, including social desirability and subjective perceptions. Future studies integrating multiple assessment methods—such as observational coding of parent–child interactions, informant reports (e.g., from children or partners), and physiological indicators—would enhance the validity and comprehensiveness of outcome evaluations. Additionally, both mentalizing and child development are dynamic, ongoing processes. Thus, future research should investigate the long-term effects of the intervention, including the potential for sustained changes in parental mentalizing and child outcomes. It will also be important to explore the provision of extended or more intensive MBT interventions for families with greater needs. As the program expands, subsequent studies may refine and scale up the intervention to include more diverse and representative populations.

In summary, as a preliminary effort to implement a MBT-based preventive interventions in a non-Western context, the present study makes three primary scientific contributions. First, it extends the existing evidence base of the LPP to Chinese parents, thereby supporting the cross-cultural robustness of its core mechanisms, such as parental mentalizing. Second, by targeting mentalization processes, the study addresses the reciprocal relationship between parenting stress and child mental health risks, with the aim of enhancing the wellbeing of both parents and children and promoting virtuous cycles in parent–child interactions. Third, the mixed-methods design and multidimensional outcome measures—encompassing parent, child, and relationship variables as well as participant perspectives—enable a comprehensive evaluation of the intervention's potential impact.
